# m6A regulator-mediated RNA methylation modification patterns are involved in the regulation of the immune microenvironment in ischaemic cardiomyopathy

**DOI:** 10.1038/s41598-023-32919-4

**Published:** 2023-04-11

**Authors:** Peng-Fei Zheng, Xiu-Qin Hong, Zheng-Yu Liu, Zhao-Fen Zheng, Peng Liu, Lu-Zhu Chen

**Affiliations:** 1 Cardiology Department, Hunan Provincial People’s Hospital, No. 61 West Jiefang Road, Furong District, ChangshaHunan, 410000 China; 2 Clinical Research Center for Heart Failure in Hunan Province, No. 61 West Jiefang Road, Furong District, Changsha, 410000 Hunan China; 3 Epidemiology Department, Hunan Provincial People’s Hospital, No. 61 West Jiefang Road, Furong District, Changsha, 410000 Hunan China; 4 Department of Cardiology, The Central Hospital of ShaoYang, No. 36 QianYuan Lane, Daxiang District, Shaoyang, 422000 Hunan China

**Keywords:** Cardiovascular diseases, Biomarkers, Cardiology, Health care, Diagnosis

## Abstract

The role of RNA N6-methyladenosine (m6A) modification in the regulation of the immune microenvironment in ischaemic cardiomyopathy (ICM) remains largely unclear. This study first identified differential m6A regulators between ICM and healthy samples, and then systematically evaluated the effects of m6A modification on the characteristics of the immune microenvironment in ICM, including the infiltration of immune cells, the human leukocyte antigen (HLA) gene, and HALLMARKS pathways. A total of seven key m6A regulators, including WTAP, ZCH3H13, YTHDC1, FMR1, FTO, RBM15 and YTHDF3, were identified using a random forest classifier. A diagnostic nomogram based on these seven key m6A regulators could effectively distinguish patients with ICM from healthy subjects. We further identified two distinct m6A modification patterns (m6A cluster-A and m6A cluster-B) that are mediated by these seven regulators. Meanwhile, we also noted that one m6A regulator, WTAP, was gradually upregulated, while the others were gradually downregulated in the m6A cluster-A vs. m6A cluster-B vs. healthy subjects. In addition, we observed that the degree of infiltration of the activated dendritic cells, macrophages, natural killer (NK) T cells, and type-17 T helper (Th17) cells gradually increased in m6A cluster-A vs. m6A cluster-B vs. healthy subjects. Furthermore, m6A regulators, including FTO, YTHDC1, YTHDF3, FMR1, ZC3H13, and RBM15 were significantly negatively correlated with the above-mentioned immune cells. Additionally, several differential HLA genes and HALLMARKS signalling pathways between the m6A cluster-A and m6A cluster-B groups were also identified. These results suggest that m6A modification plays a key role in the complexity and diversity of the immune microenvironment in ICM, and seven key m6A regulators, including WTAP, ZCH3H13, YTHDC1, FMR1, FTO, RBM15, and YTHDF3, may be novel biomarkers for the accurate diagnosis of ICM. Immunotyping of patients with ICM will help to develop immunotherapy strategies with a higher level of accuracy for patients with a significant immune response.

## Introduction

Coronary artery disease (CAD) is the leading cause of morbidity and mortality worldwide. The basic pathological changes of CAD mainly manifest as the continuous accumulation of a large amount of lipids under the intima of the coronary artery and the formation of atherosclerotic plaques. The continuous progression of atherosclerotic plaques gradually leads to the narrowing of the coronary artery lumen and finally, impairment of myocardial blood perfusion^1^. Sustained myocardial blood perfusion related damage leads to myocardial necrosis due to ischaemia and hypoxia, and eventually to ICM, and its clinical manifestations mainly include chest pain, chest tightness, decreased exercise tolerance, dyspnoea, left ventricular dysfunction, arrhythmia, and ultimately death^2^. As the pathological basis of ICM, atherosclerosis is essentially a chronic inflammatory process that involves multiple immune or inflammatory mechanisms^3^. At present, Fernandez et al. systematically expounded the infiltration of immune cells in atherosclerotic plaques for the first time and further described the different activation states of infiltrated immune cells, which paved the way for the study of the underlying mechanism of autoimmune reactions in atherosclerosis^4^. Yang et al. have suggested that monocyte infiltration increased and CD8 + T-cell infiltration decreased in patients with CAD^5^. Moreover, in a recently published study, we found that the infiltration of M0 macrophages and neutrophils increased, whereas the infiltration of CD8 + T cells, gamma delta (γδ) T cells, and resting mast cells decreased in patients with acute myocardial infarction (AMI)^6^. These results suggest that the immune mechanism plays a key role in atherosclerosis and related cardiovascular diseases. However, the exact immunomodulatory mechanism involved in ICM is still unclear. Elucidating the immune regulation mechanism of ICM may be essential to reveal the underlying pathological mechanism and may help to identify new immunotherapies for ICM.

Traditional epigenetic modification refers to the reversible modification of proteins (histones) and DNA, which can regulate gene expression without changing the genetic sequence^7^. Recently, RNA modifications have gradually attracted attention and is considered the third layer of epigenetics, through which RNA metabolism and processing can be regulated^8^. Previous studies have shown that RNA modifications exist in all identified life forms. A variety of modification forms have been found, including N1-methyladenosine (m1A), 5-methylcytosine (m5C), and N6-methyladenosine (m6A). The most common modification is m6A modifications, which is a homeostatic and reversible process in eukaryotic cells that is mainly regulated by a variety of m6A regulatory factors, including demethylases (erasers), binding proteins (readers), and methyltransferases (writers)^10^. Specifically, the process of m6A methylation is regulated by several methyltransferases, such as WTAP, METTL14 and METTL3, while the demethylation process of m6A methylation is mediated by several demethylases, including FTO and ALKBH5. In addition, readers are a group of m6A-binding proteins that can recognize the methylation motifs of m6A, which mediating the regulatory functions of m6A and belong to the YTHDF and YTHDC families^11^.

Recent studies have shown that m6A regulation may partially explain some potential molecular mechanisms of immune regulation. For example, Wang et al. found that the HNRNPA2B1 regulator could promote m6A modification and trigger an innate immune response by recognizing viral DNA during viral infection^12^. Han et al. found that YTHDF1 is involved in the antigen presentation of dendritic cells to CD8 + T cells by enhancing lysosomal cathepsin translation and impounding tumour neoantigen cross-presentation and CD8 + T-cell cross-priming, thereby promoting the immune escape of tumour cells^13^. Li et al. found that the homeostatic differentiation of T cells may be severely impaired due to the deletion of METTL3 in T cells^14^. Although increasing evidence has shown that m6A plays a key regulatory role in the immune response, none of these research studies have focused on the role of m6A in the pathogenesis of ICM. Therefore, an in-depth investigation of the immune changes between healthy subjects and ICM patients, as well as the possible changes in m6A regulators related to these changes, can deepen our understanding of the pathogenesis of ICM from a completely new perspective.

## Results

### Data pre-processing

The normalized gene expression matrix of the GSE1869, GSE5406, and GSE57338 datasets was obtained by removing outliers, standardizing the data format, and adding missing values. The integrated expression profile, which included 12,529 different gene symbols, was obtained after data merging and eliminating interbatch differences between the GSE1869 and GSE5406 datasets, and was defined as the training set (Supplementary Table S1). Then, the gene expression profile of GSE57338, which included 18,859 different gene symbols, was defined as the testing set (Supplementary Table S2A and S2B).

### The landscape of m6A regulators between healthy and ICM samples

**Figure 1:**
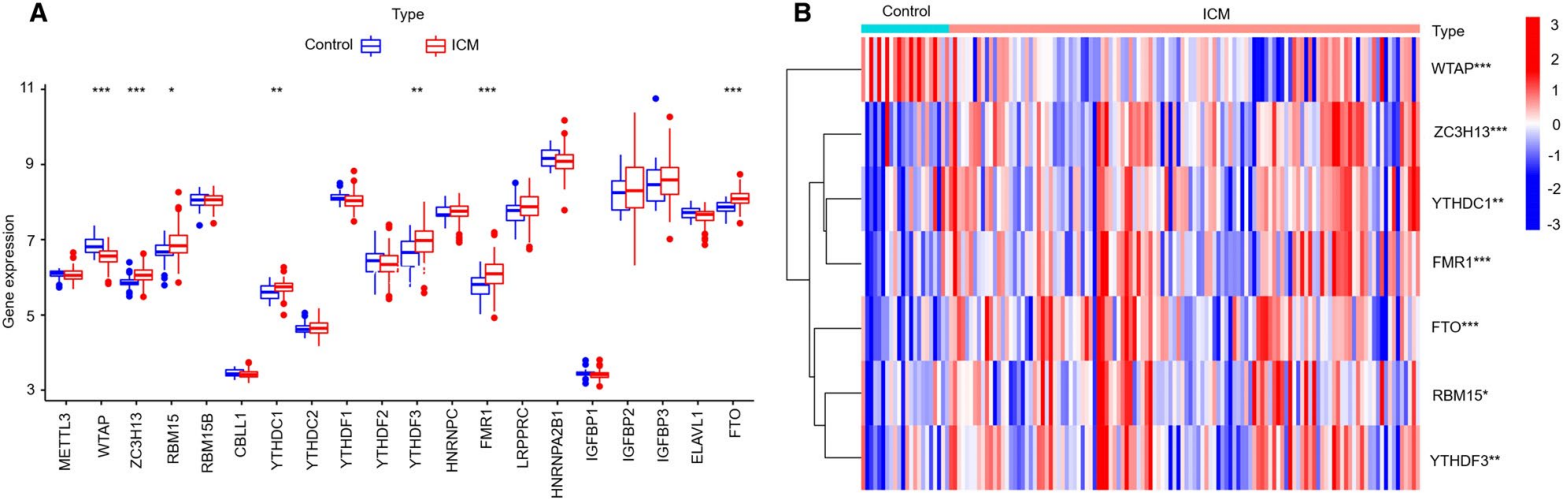
Expression landscape of m6A RNA methylation regulators in ICM. Box plot (**A**) and heatmap (**B**) showing the expression of seven m6A regulators that are significantly differentially expressed between healthy and ICM samples. *, *P* < 0.05; **, *P* < 0.01; ***, *P* < 0.001.

A total of 20 different m6A regulators, including 6 writers (METTL3, ZC3H13, RBM15, RBM15B, WTAP, and CBLL1), 13 readers (YTHDC1, LRPPRC, HNRNPC, IGFBP1, YTHDC2, HNRNPA2B1, YTHDF1, FMR1, YTHDF3, IGFBP2, YTHDF2, IGFBP3, and ELAVL1) and 1 eraser (FTO), were analysed in this research study. As shown in the box plot (Fig. 1A) and heatmap (Fig. 1B), we noted that the expression level of WTAP had decreased significantly, while the expression levels of ZC3H13, YTHDC1, FMR1, FTO, RBM15, and YTHDF3 had increased significantly in the ICM patients, compared with healthy subjects.

### Random forest screening for key m6A regulators

**Figure 2:**
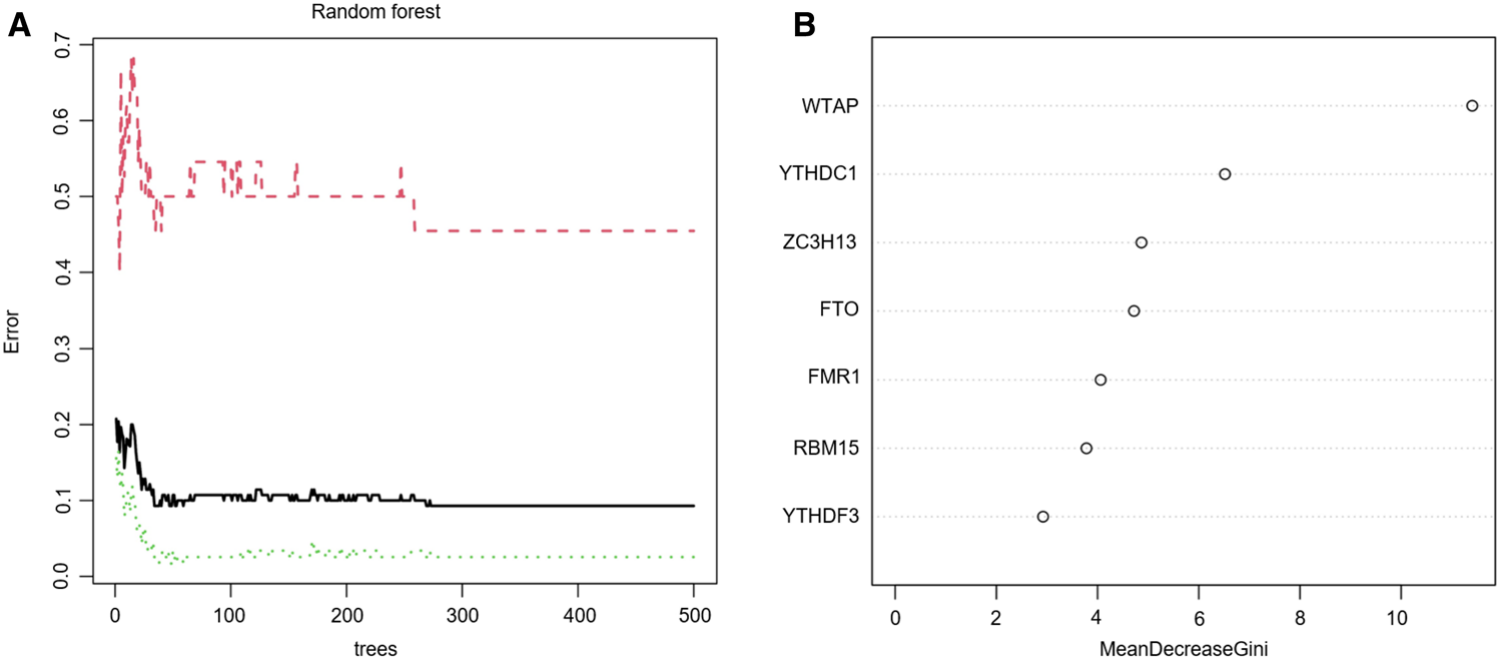
Random forest model construction to identify the key m6A regulators. (**A**) The influence of the number of decision trees on the error rate. The x-axis represents the number of decision trees, and the y-axis indicates the error rate. When the number of decision trees was approximately 300, the error rate was relatively stable. (**B**) Results of the Gini coefficient method in the random forest classifier. The x-axis indicates genetic variables while the y-axis represents the importance index.

Cyclic random forest classification was performed for all possible combinations in 1–20 variables, and the average error rate of the pattern mode was calculated. Referring to the relationship plot between the number of decision trees and the model error (Fig. 2A), 300 trees were selected as the parameter of the final model, which indicates stable error in the model. Subsequently, as shown in Fig. 2B, seven key m6A regulators (WTAP, ZC3H13, YTHDC1, FMR1, FTO, RBM15, and YTHDF3) with importance greater than 2 were selected for subsequent analysis.

### Construction and assessment of a nomogram model for the diagnosis of ICM

**Figure 3:**
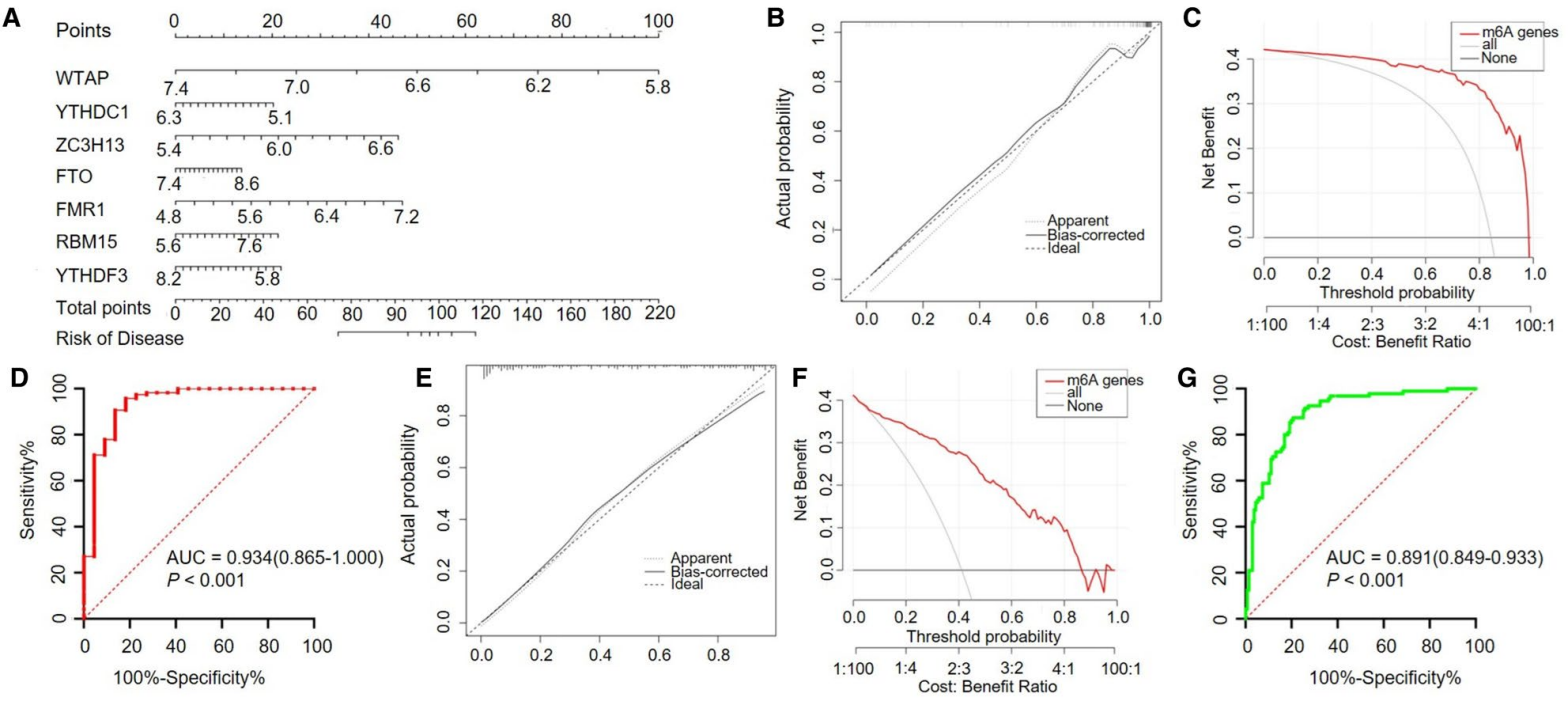
Construction and validation of a predictive nomogram for ICM established based on seven m6A regulators. (**A**) The constructed nomogram. The calibration plot, DCA and ROC analysis of the nomogram in the training set (**B**,**C**,**D**) and in the testing set (**E**,**F**,**G**). (**B**,**E**) The diagonal dotted lines represent a perfect prediction using an ideal model. (**C**,**F**) The solid lines represent the performance of the nomogram, of which a closer fit to the diagonal dotted line represents a better prediction. (**D**,**G**) ROC analysis reconfirmed that the model was effective in distinguishing ICM patients from healthy subjects.

As shown in Fig. 3A, a predictive nomogram was constructed based on the seven key m6A regulators (WTAP, ZC3H13, YTHDC1, FMR1, FTO, RBM15, and YTHDF3) in the training set (GSE1869 combined with GSE5406). The calibration curve suggests that the error between the predicted risk and the actual ICM risk was very small, indicating that the nomogram model achieved a high degree of accuracy in predicting ICM in the training set (Fig. 3B) and the testing set (Fig. 3E). Decision curve analysis (DCA) showed that the “nomogram” curve was higher than the grey line, indicating that the nomogram maintained a great level of clinical utility in predicting the morbidity of ICM patients in the training set (Fig. 3C) and testing set (Fig. 3F). ROC analysis reconfirmed that the model was effective in distinguishing ICM patients from healthy subjects in the training set (Fig. 3D) and testing set (Fig. 3G).

### m6A RNA methylation modification patterns mediated by the 7 key m6A regulators in ICM

**Figure 4:**
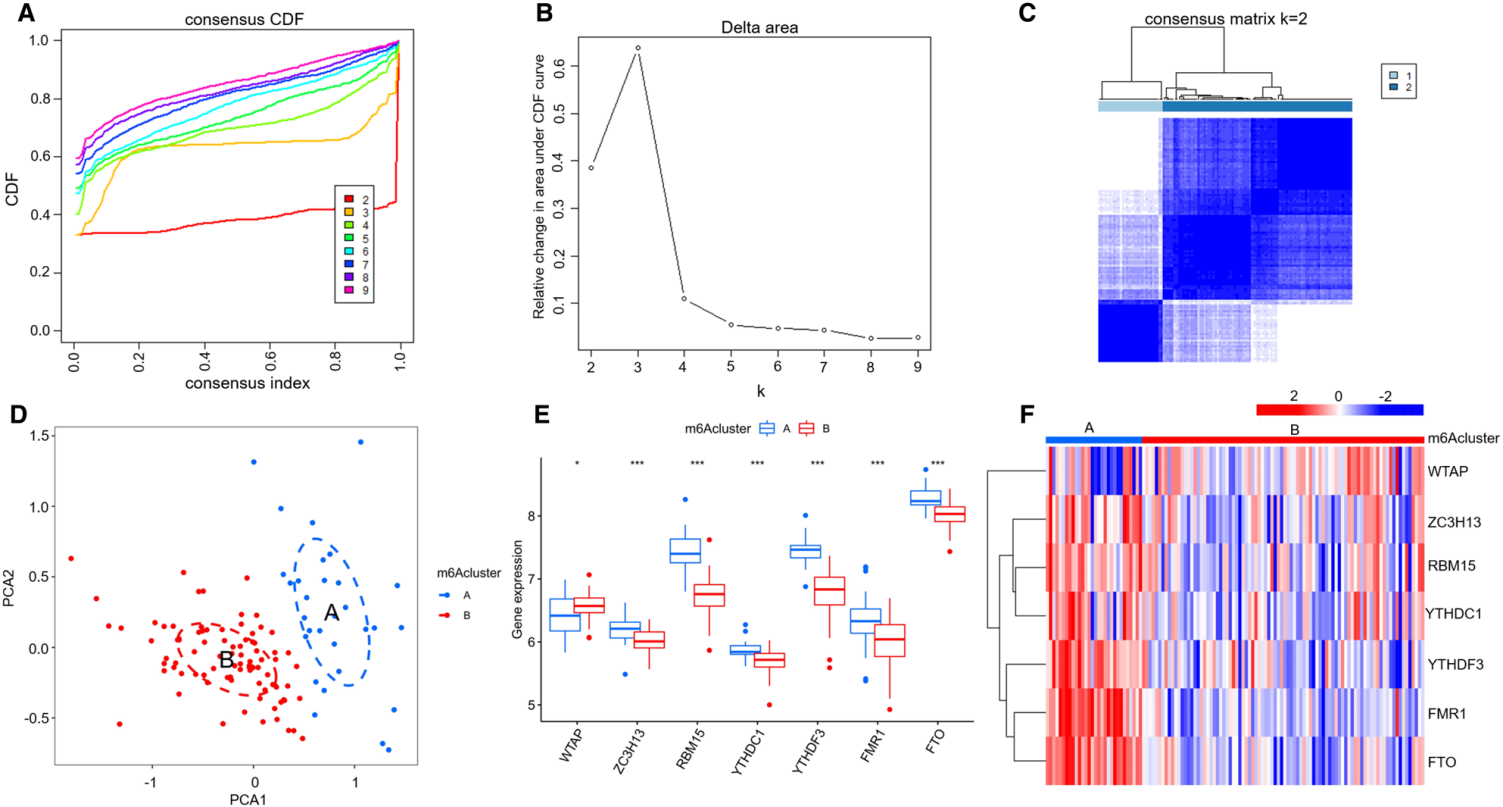
Identification of two distinct m6A modification pattern subtypes in ICM. (**A**) Consensus clustering cumulative distribution function (CDF) for k = 2–9. (**B**) Relative changes in the area under the CDF curve for k = 2–9. (**C**) Heatmap of the matrix of the co-occurrence proportions in the ICM samples. (**D**) Principal component analysis of the transcriptome profiles of the two m6A subtypes, showing a remarkable difference in the transcriptome between the different modification patterns. The box plot (**E**) and heatmap (**F**) showing that the expression of seven m6A regulators were significantly different between the m6A-cluster-A and -B groups. *, *P* < 0.05; ***, *P* < 0.001.

Unsupervised consistent clustering analysis based on the expression values of the seven key m6A regulators in the ICM samples were used to study the m6A modification patterns in ICM (Fig. 4A–C). Two different subtypes of ICM were identified based on qualitatively different expression of the seven key m6A regulators, including 30 samples in the m6A cluster-A group and 88 samples in the m6A cluster-B group (Fig. 4D, Supplementary Table S3). In addition, we noted that the expression of WTAP increased, while the expression of ZC3H13, YTHDC1, FMR1, FTO, RBM15, and YTHDF3 decreased in the m6A cluster-B group, compared with that of m6A cluster-A group (Fig. 4E,F), indicating the existence of two distinct m6A modification patterns in ICM.

### Differences in immune microenvironment characteristics between the control and ICM samples and among the two distinct m6A modification patterns

**Figure 5:**
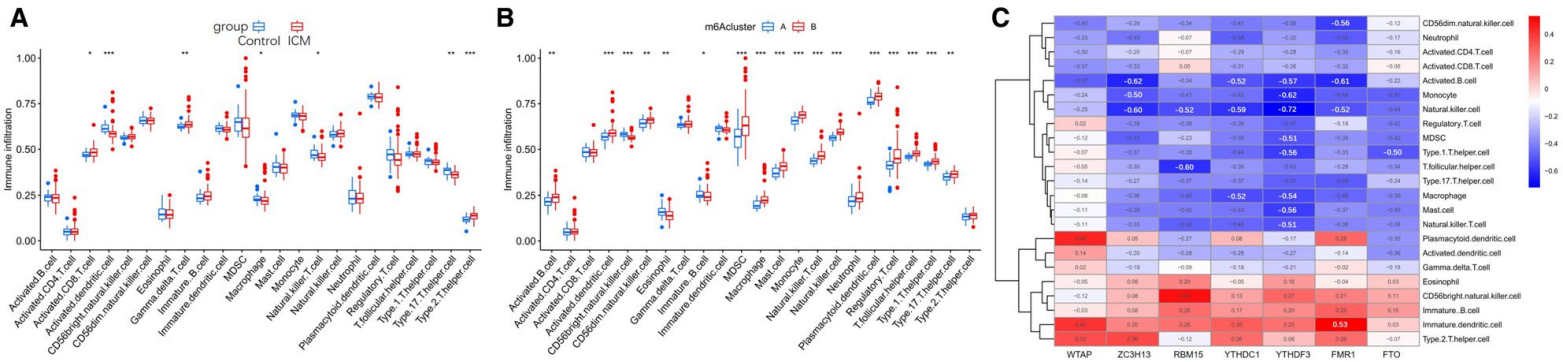
Diversity of immune microenvironment characteristics among the two distinct m6A modification patterns. The differences in the abundances of each immune microenvironment-infiltrating immunocyte between control and ICM samples (**A**); m6A cluster-A and -B (**B**). (**C**) The associations between 7 key m6A regulators and several immune cells. *, *P* < 0.05; **, *P* < 0.01; ***, *P* < 0.001.

**Figure 6:**
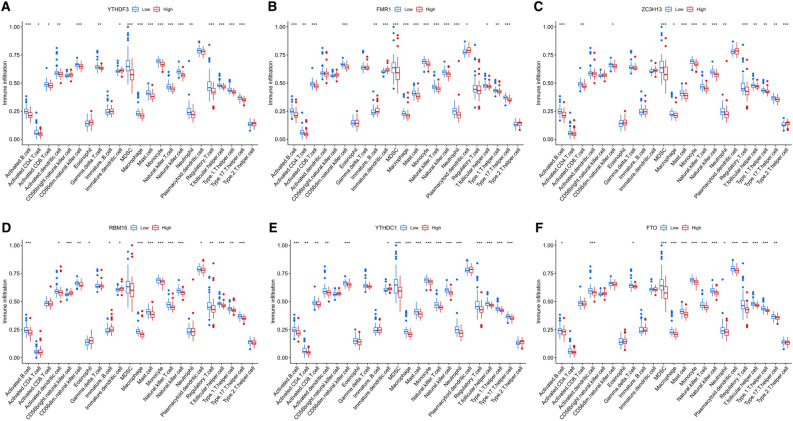
Comparison of immunocyte abundance in the high- and low-expression groups of YTHDF3 (**A**), FMR1 (**B**), ZC3H13 (**C**), RBM15 (**D**), YTHDC1 (**E**), FTO (**F**). *, *P* < 0.05; **, *P* < 0.01; ***, *P* < 0.001.

**Figure 7:**
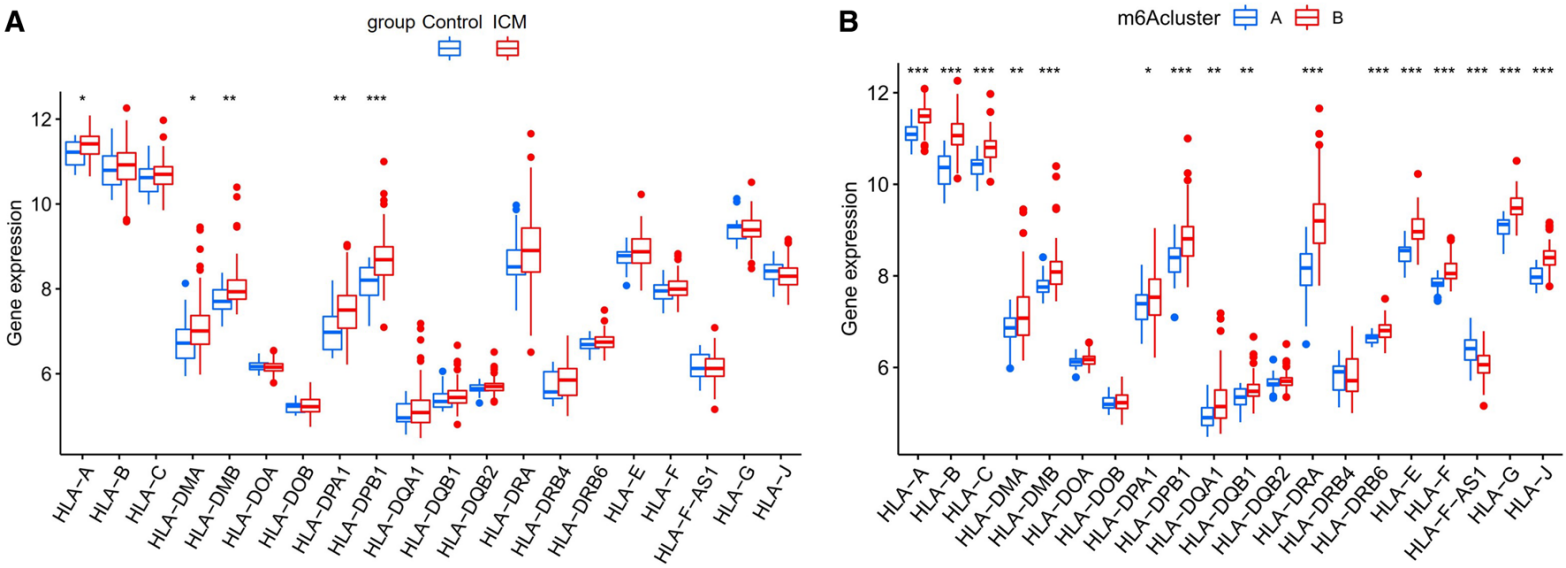
The differences in the expression levels of each HLA gene between the control and ICM groups (**A**); m6A cluster-A and -B groups (**B**). *, *P* < 0.05; **, *P* < 0.01; ***, *P* < 0.001.

The infiltration levels of many immunocytes was different between the control and ICM groups and among the two distinct m6A modification patterns. We noted a higher level of infiltration of activated CD8 T cells, gamma delta T cells, and Type-2 T helper cells, and a lower level of infiltration of activated dendritic cells, macrophages, NK T cells, and Type-17 T helper cells in the control group, compared to the ICM group (Fig. 5A). Meanwhile, we also found a relatively higher infiltration level of activated B cells, T follicular helper cells, regulatory T cells (Treg), NK T cells, NK cells, CD56dim NK cells, plasmacytoid dendritic cells, myeloid-derived suppressor cells (MDSCs), mast cells, activated dendritic cells, monocytes, macrophages, type-1 T helper cells, and type-17 T helper (Th17) cells, as well as lower infiltration levels of CD56bright NK cells, eosinophils, and immature B cells in the m6A cluster-B group, than in the m6A cluster-A group (Fig. 5B). More details on the immune cell infiltration levels in the samples are provided in Supplementary Table S4. As shown in Fig. 5C, we noted that ZC3H13 expression was significantly negatively correlated with the numbers of activated B cells (r = − 0.62), monocytes (r = − 0.50), and NK cells (r = − 0.60); RBM15 expression was significantly negatively correlated with the numbers of T follicular helper cells (r = − 0.60) and NK cells (r = − 0.52); YTHDC1 expression was significantly negatively correlated with the numbers of activated B cells (r = − 0.52), NK cells (r = − 0.59), and macrophages (r = − 0.52); YTHDF3 expression was significantly negatively correlated with the numbers of monocytes (r = − 0.62), activated B cells (r = − 0.57), MDSCs (r = − 0.51), Type-1 T helper cells (r = − 0.56), macrophages (r = − 0.54), mast cells (r = − 0.56), NK T cells (r = − 0.51), and NK cells (r = − 0.72); FMR1 expression was significantly negatively correlated with the numbers of CD56dim NK cells (r = − 0.56), activated B cells (r = − 0.61), and NK cells (r = − 0.52); while FTO expression was significantly negatively correlated with the number of Type-1 T helper cells (r = − 0.50) (*P* < 0.05 for all). Moreover, compared with the low YTHDF3 expression group, the numbers of infiltrated activated B cells, activated dendritic cells, activated CD4 T cells, CD56dim NK cells, activated CD8 T cells, gamma delta T cells, MDSCs, macrophages, mast cells, monocytes, NK T cells, neutrophils, NK cells, Treg cells, type-1 T helper cells, T follicular helper cells, and Th17 cells were low, while the number of infiltrated immature dendritic cells were high in the high YTHDF3 expression group (Fig. 6A). Similar patterns of immune cell infiltration were found between the high and low expression groups of FMR1 (Fig. 6B), ZC3H13 (Fig. 6C), RBM15 (Fig. 6D), YTHDC1 (Fig. 6E), and FTO (Fig. 6F). In addition, we noted that the expression levels of HLA-A, HLA-DMA, HLA-DMB, HLA-DPA1, and HLA-DPB1 were significantly elevated in ICM patients than in control subjects (Fig. 7A). We also found that the expression levels of HLA-DMA, HLA-A, HLA-DQA1, HLA-B, HLA-DPA1, HLA-C, HLA-DMB, HLA-E, HLA-DQB-1, HLA-DRB6, HLA-DRA, HLA-F, HLA-J, HLA-G, and HLA-DPB1 were significantly elevated, while the expression level of HLA-F-AS1 was significantly downregulated in the m6A cluster-B group, compared with the m6A cluster-A group (Fig. 7B).

### Biological characteristics of healthy subjects and ICM patients

**Figure 8:**
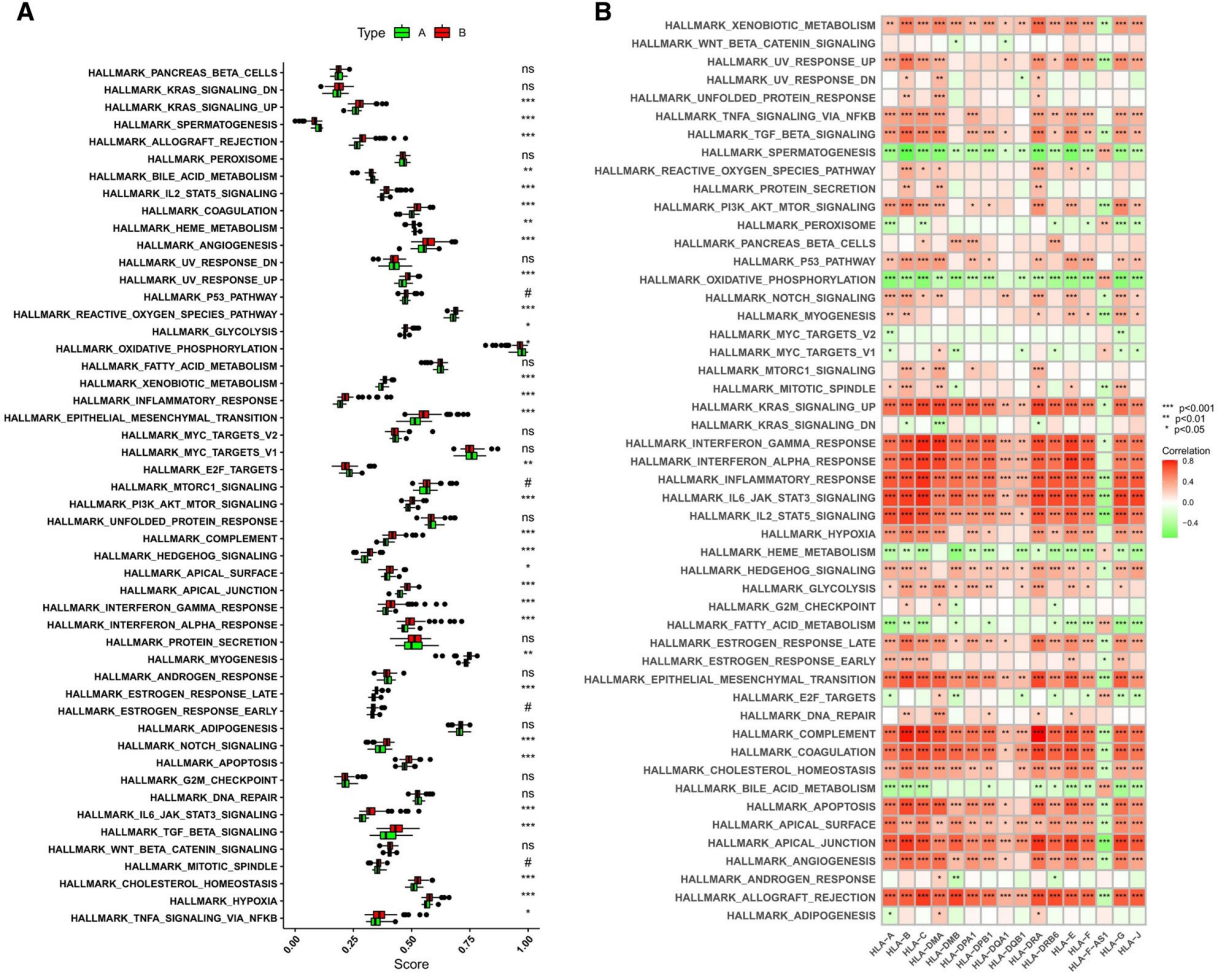
The underlying diversity in the biological functional characteristics among the two m6A modification patterns. (**A**) Differences in HALLMARKS pathway enrichment scores between m6A cluster-A and -B. (**B**) Heatmap showing the correlations between HLA genes and 50 HALLMARKS pathways. *, *P* < 0.05; **, *P* < 0.01; ***, *P* < 0.001.

The HALLMARKS pathways between healthy subjects and ICM patients were compared via gene set variation analysis (GSVA). Compared with the m6A cluster-A group, the m6A cluster-B group had a larger number of enriched pathways, such as the TNF-a signalling pathway via NF-kB, apoptosis, the apical junction, the apical surface, cholesterol homeostasis, the late estrogen response, the interferon gamma response, glycolysis, IL-6/JAK/STAT3 signalling pathway, the interferon alpha response, UV response up, myogenesis, hypoxia, TGF-β signalling, epithelial mesenchymal transition, notch signalling, hedgehog signalling pathway, PI3K/Akt/mTOR signalling pathway, inflammatory response, xenobiotic metabolism, the reactive oxygen species pathway, angiogenesis, coagulation, IL-2/STAT5 signalling pathway, allograft rejection, complement, and KARS signalling up, as well as poorly enriched pathways, including E2F targets, oxidative phosphorylation, haem metabolism, bile acid metabolism, and spermatogenesis (Fig. 8A, Supplementary Table S5). In addition, we also noted that the HLA subtype genes, except for HLA-F-AS1, were positively correlated with xenobiotic metabolism, the interferon alpha response, KRAS signalling pathway, the interferon gamma response, IL-2/STAT5 signalling, the inflammatory response, IL-6/JAK/STAT3 signalling pathway, epithelial mesenchymal transition, coagulation, complementation, the apical surface, the apical junction, and allograft rejection, and was negatively associated with spermatogenesis, while HLA-F-AS1 expression was negatively associated with some of the pathways indicated above (Fig. 8B).

### Effect of m6A modification on the immune microenvironment in ICM

**Figure 9:**
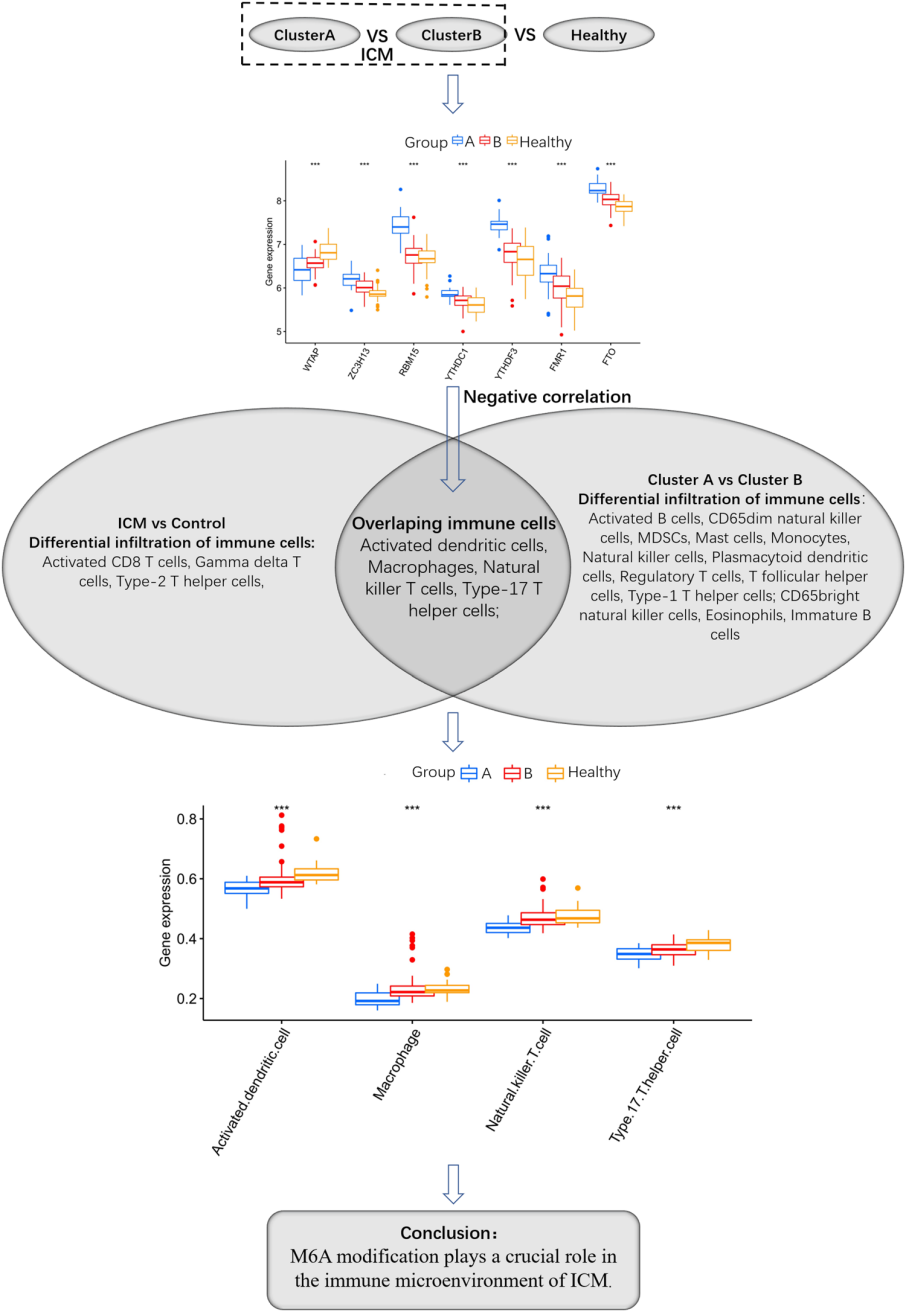
The summary diagram. ***P < 0.001.

As shown in Fig. 9, the infiltration levels of immune cells, such as activated dendritic cells, macrophages, NK T cells, and Th17 cells, were found to differ between control subjects and ICM patients, and between two different m6A modification patterns in ICM. WTAP, a m6A regulator, was found to be progressively upregulated, while the other six m6A regulators, ZCH3H13, YTHDC1, FMR1, FTO, RBM15, and YTHDF3, were gradually downregulated in the m6A cluster-A vs. m6A cluster-B vs. healthy subjects. Additionally, the m6A regulators: FTO, YTHDC1, YTHDF3, FMR1, ZC3H13, and RBM15 were found to be significantly negatively correlated with the aforementioned immune cells. Moreover, it was observed that the infiltration of activated dendritic cells, macrophages, NK T cells, and Th17 cells were gradually increased in the m6A cluster-A vs. m6A cluster-B vs. healthy subjects.

## Discussion

Recently, ICM has become the leading cause of heart failure. Increasing evidence shows that innate immune mechanisms play a crucial role in the occurrence and development of myocardial ischaemia and heart failure. Mild to moderate innate immune responses may limit the extent of heart damage and promote the repair of cardiac function, whereas dysregulated immune response is likely to be deleterious^15^. Accumulating evidence has indicated that m6A modifications play an integral role in innate and adaptive immune responses^16^. A large number of studies have explored the role of m6A modifications in immunity, especially in the infiltration of immune cells in the tumour microenvironment, and the results have confirmed that m6A modifications play a fundamental role in tumour immunity^17,18^. Therefore, we believe that m6A modifications play a similar role in shaping the immune microenvironment in ICM. To better elucidate these issues, the effect of m6A modifications on the characteristics of the immune microenvironment in ICM was explored.

In this study, the effects of m6A modifications on immune cell infiltration, the expression of HLA subtype genes, inflammation, and immune-related pathways in ICM were explored, and several meaningful novel findings were made. First, we noted that the expression levels of seven m6A regulators, including WTAP, YTHDC1, ZC3H13, FTO, FMR1, RBM15, and YTHDF3 were significantly different between ICM patients and healthy subjects. Second, we identified two distinct m6A modification patterns (m6A cluster-A and -B) through the unsupervised clustering of ICM samples based on the expression values of seven key m6A regulators. Third, we noted that the expression of one m6A regulator, WTAP, was gradually elevated, while the expression levels of the other 6 m6A regulators, ZCH3H13, YTHDC1, FMR1, FTO, RBM15, and YTHDF3, were gradually downregulated in the m6A cluster-A vs. m6A cluster-B vs. healthy subjects. We also found that the infiltration levels of the activated dendritic cells, macrophages, natural killer (NK) T cells, and type-17 T helper (Th17) cells, gradually increased in the m6A cluster-A vs. m6A cluster-B vs. healthy subjects. Meanwhile, the m6A regulators, FTO, YTHDC1, YTHDF3, FMR1, ZC3H13, and RBM15, were significantly negatively correlated with the above-mentioned immune cells. These findings suggest that m6A modifications can significantly affect the immune microenvironment in ICM, and that m6A modifications, immune cell infiltration, and their interactions play a crucial role in the occurrence and development of ICM. This classification strategy of immune subtypes can help us subtype ICM samples at the immune level or molecular level to help us better understand the underlying mechanisms of immune regulation in ICM. A recent study used this approach to identify two distinct m6A modification patterns in AMI, and the results of that study helped us enhance our understanding of the immune microenvironment in AMI^19^. At present, molecular subtyping strategies have been widely used in tumour research, and the identification of novel molecular subtypes can help to formulate better treatment plans^20,21^. Therefore, the further elucidation of the potential mechanisms of m6A modifications in regulating various infiltrating immune cells can help develop new therapeutic strategies for ICM.

Previous studies have shown that lymphocytes, monocytes/macrophages, and mast cells may also contribute to myocardial fibrosis by secreting several key fibrogenic mediators, including mast cell-derived proteases, matricellular proteins, chemokines, inflammatory cytokines, endothelin-1, and growth factors^22^. Recent studies have revealed that during predominant type of macrophages is the M1 subtype in the infarction subtype during the early stages of myocardial infarction (2–5 days). These macrophages are capable of damaging the myocardium by releasing reactive oxygen species, inflammatory mediators, and proteases. However, during the late stages of infarction, macrophages in the affected area are mostly of the M2 subtype, which can improve cardiac function by reducing cardiac fibrosis and myocardial remodeling caused by ischemia–reperfusion injury^23^. Anzai et al. found that the expression levels of interleukin-1β (IL-1β), IL-18, and tumour necrosis factor-α (TNF-α) were significantly elevated in mice with dendritic cell ablation, compared with the control group. These mice also showed deterioration of left ventricular function and ventricular remodelling, and suggested that dendritic cells play an important role in regulating monocyte/macrophage homeostasis and, therefore, act as a immunoprotective modulators in post-infarction healing^24^. Forte et al. noted that the activation of cytotoxic CD8 T cells by dendritic cells contributes to the aggravation of inflammatory injury and the corresponding decline in cardiac function following myocardial ischaemia^25^. Backteman et al. have suggested that the infiltration of NK cells was significantly reduced in CAD and ICM patients^26^, while Ong et al. found that NK cells can delay the development of cardiac fibrosis both by directly reducing collagen formation in cardiac fibroblasts and by inhibiting the accumulation of specific inflammatory mediators in the heart^27^. In this study, we also found that several immune cells, including activated dendritic cells, macrophages, and NK T cells, exert a protective role in the occurrence and development of ICM, while activated CD8 T cells play a role in promotion. These findings are generally consistent with a results of a previous study. In addition, previous studies have shown that the antiangiogenic and profibrotic functions of type-17 T helper (Th17) and dysfunctional regulatory T (Treg) cells play an indispensable role during the progression into ischaemic heart failure^28,29^. However, we noted that Th17 cells may also play a protective role in the development and progression of ICM, whereas Th2 cells play an opposing role during this pathological process. These findings indicate that immune cell infiltration plays a key role in the development of ICM, and its related mechanisms are complex and diverse, while more in vitro and in vivo studies are needed to further clarify the results of this study.

Human major histocompatibility complex (MHC), also known as human leukocyte antigen (HLA), is encoded by the HLA gene complex. HLAs can be divided into class I, II and III antigens based on their function and distribution. HLA-A, -B and HLA-C are classical HLA class I antigens; HLA-DQ, -DP and -DR are classical HLA class II antigens; HLA‐X, -H, -E, -DO, -G, -F, -DM, and -DN are non-classical HLA class I and II molecules; while other antigens, such as complement, are class III antigens^30,31^. HLAs are significantly associated with the regulation, monitoring, and immune response and plays a crucial role in autoimmune diseases, tumour immunity, and reproductive immunity^32^. Several studies have attempted to associate dilated cardiomyopathy, ICM, or heart failure with specific HLAs. McKenna et al. noted that HLA-DR4 showed the most significant correlation with dilated cardiomyopathy^33^. Osa et al. found that the presence of HLA-B‐15 and HLA-DQ3 was associated with advanced dilated cardiomyopathy, while the absence of HLA-A1, HLA-B8, and HLA-DQ2 are associated with the development of severe ICM. Almasood et al. noted that HLA-G is upregulated in patients with heart failure, and that serum HLA-G may be a potential new biomarker with a higher degree of sensitivity than other classical biomarkers of heart failure^34^. However, it is still unclear whether there are differences in the expression of HLA genes between control subjects and ICM patients, as well as among the two different molecular subtypes of ICM. We noted that the expression levels of HLA-A, HLA-DMA, HLA-DMB, HLA-DPA1, and HLA-DAB1 were significantly elevated in ICM patients, compared with control subjects. Meanwhile, we also found that the expression levels of HLA-C, HLA-DPA1, HLA-J, HLA-DMA, HLA-E, HLA-DQA1, HLA-A, HLA-DQB-1, HLA-F, HLA-DPB1, HLA-DMB, HLA-B, HLA-DRB6, HLA-G, and HLA-DRA were significantly upregulated, while the expression level of HLA-F-AS1 was significantly downregulated in the m6A cluster-B group, compared with the m6A cluster-A group. In addition, compared with the m6A cluster-A group, the m6A cluster-B group was more enriched in inflammatory or immune-related signalling pathways, including TGF-β signalling, TNF-α signalling via NF-kB, Notch signalling, IL-2/STAT5 signalling, IL-6/JAK/STAT3 signalling, PI3K/Akt/mTOR signalling, hedgehog signalling, and KARS signalling. Meanwhile, some associations between HLA subtype genes and these inflammatory- or immune-related pathways were also noted. These results suggest that immune or inflammatory activity is also different among the two different m6A modified subtypes of ICM. Immunotyping of ICM will help identify samples with a significant immune response, which will contribute to more precise immune intervention.

This research study has several limitations. First, the immune cell analysis in this study adopts the most widely used analysis method to quantify the number of immune cells, but single-cell sequencing is still required to obtain the most accurate number of immune cells. Second, we were unable to obtain additional clinical features or serological results of the ICM samples in the GSE1869, GSE5406, and GSE57338 datasets. Therefore, it is difficult to determine the key role played by m6A modifications in immune regulation from multiple perspectives and to evaluate the impact of different m6A modification patterns on the clinical outcome of patients, so the current analysis results are relatively singular. Third, this research study was based on bioinformatics analysis, and many of the findings are theoretically valid. However, the results of our study need to be verified by conducting more in vitro and in vivo experiments.

## Conclusion

This research study suggests that m6A modifications plays a key role in the complexity and diversity of the immune microenvironment of ICM. Seven key m6A regulators, including WTAP, ZCH3H13, YTHDC1, FMR1, FTO, RBM15, and YTHDF3, may be novel biomarkers for the accurate diagnosis of ICM. Immunotyping of patients with ICM can help develop immunotherapy strategies with a higher level of accuracy for patients with a significant immune response.

## Materials and methods

### ICM microarray datasets

Referring to the results of previous studies, the integration of different gene expression profile data for analysis is a reasonable approach used to identify novel key molecular targets^35,36^. Thus, a total of three microarray datasets were downloaded from the Gene Expression Omnibus (GEO, http://www.ncbi.nlm.nih.gov/geo). After normalization and elimination of interbatch differences between GSE1869 (including 6 healthy subjects and 10 ICM patients) and GSE5406 (including 108 ICM patients and 16 healthy subjects), an integrated gene expression profile was obtained and defined as the training set. Meanwhile, the gene expression matrix of GSE57338 (including 136 ICM patients and 95 healthy subjects) was defined as the testing set. All analyses included in this study were conducted using R software^37^. Specifically, the *normalize Between Arrays* function in the "*limma*" package^38^ was used to normalize the gene expression profiles of the GSE1869, GSE5406, and GSE57338 datasets. When a probe corresponded with multiple genes, it was excluded from the analysis. When multiple probes corresponded with the same gene, the average gene expression value detected by those probes was taken as the true expression value of the gene. Then, the ComBat function in the "sva" package^39^ was used to eliminate the inter-batch differences between the GSE1869 and GSE5406 datasets. Since our study re-utilized publicly available datasets, including GSE1869^40^, GSE5406^41^, and GSE57338^42^, which had been previously approved by local ethics committees during the original study, our research did not require additional ethical approval.

### Identification of key m6A regulators

The Wilcoxon test was used to evaluate the differences in the expression status of the 20 m6A regulators between healthy individuals and ICM patients. Then, a random forest model was constructed using the “randomForest” package in R software and was used to identify key m6A regulatory factors. Specifically, the average model error rate of all m6A regulatory factors were calculated, and the optimal number of variables of the binary tree in the node was set at 6, and 300 was selected as the optimal number of trees contained in the random forest. Then, the random forest model was constructed using the decreasing precision method (Gini coefficient method) and was used to obtain the dimension importance value from the random forest model. Factors with importance values greater than 2 were selected as key m6A regulators for subsequent model construction.

### Construction and verification of the nomogram

The predictive nomogram was constructed using the "rms" package in R software and was based on the expression values of the seven key m6A regulators in the training set (GSE1869 combined with GSE5406). Then, the calibration curve was used to assess the predictive power of the nomogram model. Decision curve analysis was used to evaluate the clinical value of the nomogram model. Finally, receiver operating characteristic (ROC) analysis was used to evaluate the diagnostic performance of the nomogram model in distinguishing ICM patients from healthy subjects. In addition, we also verified the predictive value of the nomogram model by constructing the calibration curve and performing decision curve analysis and ROC analysis on the external testing set (GSE57338).

### Identification of the m6A modification pattern

Based on the expression of seven key m6A regulators, unsupervised clustering analysis was used to identify different m6A modification patterns in ICM. The robustness and cluster numbers were calculated using the consensus clustering algorithm^43,44^. The R package "ConsensuClusterPlus" was used to perform the above mentioned steps for 1000 iterations to guarantee the robustness of the classification^45^. Principal component analysis (PCA) was used to further verify the different m6A modification patterns distinguished by the seven key m6A regulators. The Kruskal test was used to compare the expression levels of the 7 key m6A regulators among two distinct m6A modification patterns. Correlations among several HLA gene subtypes and between the seven key m6A regulators and immunocyte fractions were evaluated using Spearman correlation analysis.

### Single-sample gene set enrichment analysis (ssGSEA) and gene set variation analysis (GSVA) enrichment analysis

Single-sample gene-set enrichment analysis (ssGSEA) was used to estimate the number of specific infiltrating immune cells in the ICM samples, which defines an enrichment score that is representative of the degree of absolute enrichment of a gene set in each sample within a given dataset^46^. The gene list of infiltrating immunocyte gene sets was obtained from a previous study^43^. The Wilcoxon test was used to compare enrichment scores that represented immunocyte abundance between the different m6A modification patterns. Meanwhile, a total of 118 ICM samples were divided into low and high expression groups, according to the expression value of each key m6A regulator, which is significantly associated with infiltrating immune cells. Then, the abundance scores of infiltrating immune cells between the two different groups were also compared using the Wilcoxon test. Spearman correlation analysis was used to evaluate the correlation between the seven key m6A regulators and immunocyte fractions.

Gene set variation analysis (GSVA) was used to evaluate the 50 HALLMARK pathways among the two distinct m6A modification patterns using the ‘GSVA’ package in R software. The gene sets of ‘h.all.v7.0.symbols’ were extracted from the MSigDB database (http://software.broadinstitute.org/gsea/msigdb/index.jsp) to perform the GSVA. In addition, the Kruskal test was used to evaluate the expression of HLA genes among the two distinct m6A modification patterns. Then, correlations among HLA genes as well as between HLA genes and 50 HALLMARK pathways were determined using Spearman correlation analysis.

## Supplementary Information

Supplementary Information 1.

Supplementary Information 2.

Supplementary Information 3.

Supplementary Information 4.

Supplementary Information 5.

Supplementary Information 6.

Supplementary Information 7.

Supplementary Information 8.

## Data Availability

The datasets generated and/or analysed during the current study are available in the [GEO, http://www.ncbi.nlm.nih.gov/geo] repository, [GSE1869, GSE5406 and GSE57338].

## Abbreviations

GEO: Gene expression omnibus
m6A: RNA N6-methyladenosine
ICM: Ischemic cardiomyopathy
HLA: Human leukocyte antigen
CAD: Coronary artery disease
AMI: Acute myocardial infarction
γδ: Gamma delta
PCA: Principal component analysis
m5C: 5-Methylcytosine
m1A: N1-methyladenosine
ssGSEA: Single-sample gene-set enrichment analysis
GSVA: Gene set variation analysis
DCA: Decision curve analysis
ROC: Receiver operating characteristic
MDSC: Myeloid-derived suppressor cell
MMP: Matrix metalloproteinase
Treg: Regulatory T
TNF-α: Tumor necrosis factor-α
Th17: Type-17 T helper
IL: Interleukin
MHC: Major histocompatibility complex
