# Supplementary material for: m6A regulator-mediated RNA methylation modification patterns are involved in the regulation of the immune microenvironment in ischaemic cardiomyopathy
Source: Sci Rep. 2023 Apr 11;13:5904. doi: 10.1038/s41598-023-32919-4 (PMC10090050; doi:10.1038/s41598-023-32919-4)
Supplement: Supplementary file 1 — Supplementary Information 1. [file 41598_2023_32919_MOESM1_ESM.docx]

**m6A regulator-mediated RNA methylation modification patterns are involved in the regulation of the immune microenvironment in ischaemic cardiomyopathy**

Peng-Fei Zheng^1,2,3^, Xiu-Qin Hong^2,3^, Zheng-Yu Liu^1,2,3^, Zhao-Fen Zheng^1,2,3^, Peng Liu^4^ ID and Lu-Zhu Chen^4^ ID

Correspondence to: Prof. Lu-Zhu Chen and Peng Liu

E-mail: luzhuchen@163.com and ying_lpxm@163.com

ID: 0000-0001-8206-8153 and 0000-0002-3095-0352

^1^Cardiology Department, Hunan Provincial People's Hospital, No.61 West Jiefang Road, Furong District, Changsha 410000, Hunan, China

^2^Clinical Research Center for Heart Failure in Hunan Province, No.61 West Jiefang Road, Furong District, Changsha 410000, Hunan, China

^3^Epidemiology Department, Hunan Provincial People's Hospital, No.61 West Jiefang Road, Furong District, Changsha 410000, Hunan, China

^4^Department of Cardiology, The Central Hospital of ShaoYang, No.36 QianYuan lane, Daxiang District, Shaoyang 422000, Hunan, China

**Supplementary Figure 1.** Normalized for all the samples. **Figure 1A** shows GSE1869 combined with GSE5406, and **Figure 1B** shows GSE57338.

**Supplementary Table 1.** Gene expression profile of 12529 different gene symbols in the training set (GSE1869 combined with GSE5406).

**Supplementary Table 2A.** Gene expression profile of 10000 different gene symbols in the testing set (GSE57338).

**Supplementary Table 2B.** Gene expression profile of the remaining 8859 different gene symbols in the testing set (GSE57338).

**Supplementary Table 3.** Two different subtypes of ICM were identified in the training set based on qualitatively different expression of seven key m6A regulators.

**Supplementary Table 4.** The results of single-sample gene set enrichment analysis.

**Supplementary Table 5.** The results of gene set variation analysis enrichment analysis
