# Supplementary material for: m6A regulator-mediated RNA methylation modification patterns are involved in the regulation of the immune microenvironment in ischaemic cardiomyopathy
Source: Sci Rep. 2023 Apr 11;13:5904. doi: 10.1038/s41598-023-32919-4 (PMC10090050; doi:10.1038/s41598-023-32919-4)
Supplement: Supplementary file 2 — Supplementary Information 2. [file 41598_2023_32919_MOESM2_ESM.docx]

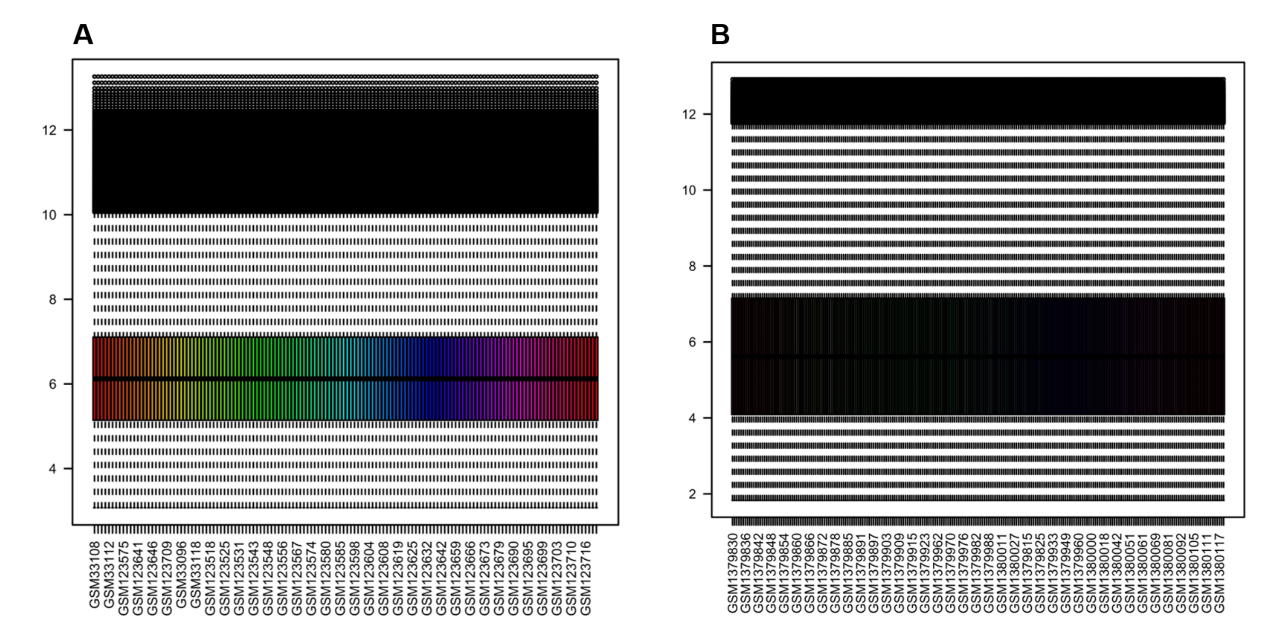


**Supplementary Figure 1**: Normalized for all the samples. **Figure 1A** shows GSE1869 combined with GSE5406 and **Figure 1B** shows GSE57338.
